# Epidemiological characteristics of human-infective RNA viruses

**DOI:** 10.1038/sdata.2018.17

**Published:** 2018-02-20

**Authors:** Mark E. J. Woolhouse, Liam Brierley

**Affiliations:** 1 Centre for Immunology, Infection and Evolution, University of Edinburgh, Ashworth Laboratories, Charlotte Auerbach Road, Edinburgh EH9 3FL, UK

**Keywords:** Infectious diseases, Epidemiology, Virology, Viral epidemiology, Viral transmission

## Abstract

RNA viruses are a major threat to human health. Here, based on extensive literature searches carried out over a period of 18 years, we provide a catalogue of all 214 known human-infective RNA virus species. We link these viruses to metadata for a number of traits that influence their epidemiology, including the date of the first report of human infection, transmissibility in human populations, transmission route(s) and host range. This database can be used in comparative studies of human-infective RNA viruses to identify the characteristics of viruses most likely to pose the greatest public health threat, both now and in the future.

## Background and Summary

Infections due to RNA viruses such as influenza virus, measles virus, HIV-1 and rotavirus impose a huge public health burden, particularly in lower and middle income countries^1^. In addition, RNA viruses are very prominent among the emerging infectious diseases: SARS coronavirus, Ebola virus and MERS coronavirus are recent, high profile examples^2^.

Our research group first published a catalogue of human-infective RNA viruses (and other infectious agents) in 2001^3^. Since then we have regularly refined our methodology and updated the catalogue as new human-infective RNA viruses are recognised and reported in the scientific literature^4,5^. In addition to identifying human-infective viruses, we record a number of traits including the date of the first report of human infection, transmissibility in human populations, transmission route(s) and host range (all fully described below).

This catalogue and metadata have been used in a number of ways. First, to estimate the diversity of human-infective RNA viruses using two techniques, extrapolation of the virus discovery curve^4^ and an ecological diversity measure^6^. Second, they have been used to identify traits associated with newly emerging viruses^3,5^. Third, they have been used to identify RNA viruses with future epidemic potential in human populations^2^. Chikungunya virus, Ebola virus and Zika virus all met the criteria prior to their actual emergence in 2005, 2014 and 2015 respectively. It has also contributed to the World Health Organisation’s Blueprint for R&D preparedness and response to public health emergencies due to highly infectious pathogens^7^.

The catalogue and metadata are subject to continual change, not only as new, human-infective RNA viruses are recognised or discovered but also as new information about their transmission routes, host range and other traits is reported in the scientific literature. The catalogue can also change retrospectively as taxonomies are revised. Here, we present a snapshot of information available as of 21st July 2017, insofar as we have been able to ascertain it using the methodology described below. We provide a list of human-infective RNA viruses, cataloguing species currently recognised by the International Committee for the Taxonomy of Viruses (ICTV^8^), and link this to metadata on seven different virus traits. We describe the distribution of virus species by discovery date, transmissibility in human populations, non-human host range, and transmission route below.

The database lists 214 ICTV-recognised, human-infective RNA virus species (Data Citation 1). This is a substantial increase on previously published counts^6^, reflecting the rapid rate of discovery and recognition of RNA viruses in recent years. These 214 species are classified in 55 genera that are members of 21 families (with one genus, *Deltavirus*, not yet assigned to a family).

**Figure 1:**
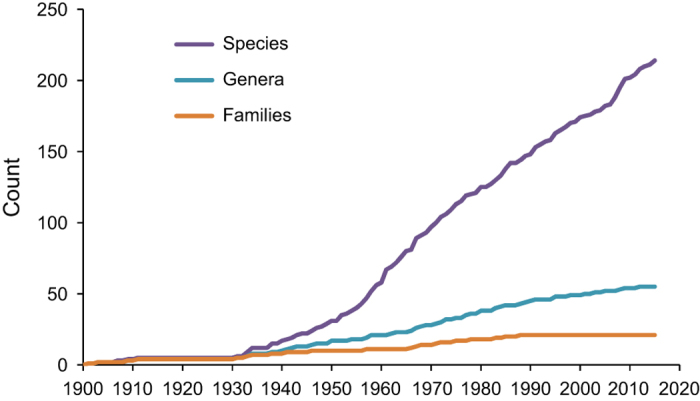
Accumulation through time of species (purple line), genera (blue) and families (orange) of RNA viruses known to infect humans.

The accumulation of (currently) ICTV-recognised, human-infective RNA virus species up to 2015 is depicted in Fig. 1. The figure goes back to 1901, when the discovery of the first human virus species–*Yellow fever virus*–was reported. The number of species increases slowly up to the mid 1950s and somewhat faster thereafter. Methods are available for extrapolating from this curve to predict future rates of discovery and the total diversity of human-infective RNA virus species^4^. We note that there is often a lag of several years between a novel virus being reported to infect humans and that virus being recognised as a new species, or not, by ICTV. As of July 2017 there were more than 20 putative RNA virus species in that category. For this reason we extend Fig. 1 only to the end of 2015. Figure 1 also depicts the accumulation of (currently) ICTV-recognised RNA virus genera and families known to contain human-infective species.

Each virus is classified according to its known level of transmissibility in human populations. Transmission may be via a natural route, including by arthropod vectors, or as unintentional iatrogenic transmission, but deliberate laboratory exposures are excluded. In keeping with previous usage^2,9^, transmissibility is assigned to one of four levels. Level 2 indicates human infective but not transmissible (between humans). Level 3 indicates transmissible from one human to another (by any natural route including arthropod vectors), but so far restricted to self-limiting outbreaks. Level 4 indicates viruses that are endemic in human populations or have the potential to cause major epidemics. Level 4 viruses are divided into 4a and 4b. Level 4a viruses are known to naturally infect non-human hosts; Level 4b viruses are known only to infect humans. Where virus species include subtypes known to have different levels of transmissibility, e.g. influenza A, the highest level is assigned. The distribution of species by these different levels of transmissibility is given in Table 1.

For each virus, we record the known range of non-human hosts, categorised as follows: non-human primates; other mammals (apart from primates); birds; reptiles; and fish. The distribution of virus species by host category is shown in Table 2. Only 7 species (3%) also infect ectotherms; and just 37 (17%) infect birds. There are 26 virus species (12%) that are only known to infect humans.

For each virus, we record all known transmission routes, categorised as follows and fully defined in the Methods section: inhalation; ingestion; sexual contact; any form of direct physical contact; fomites; bites/broken skin; iatrogenic; vector (biting arthropod); maternal. The distribution of virus species by transmission route is shown in Table 3. It is noteworthy that 91 (43%) of species can be transmitted by vectors (invariably a fly or tick).

We hope that this database will be of value to researchers undertaking comparative studies of human-infective RNA viruses, as well as being a useful reference for those interested in pathogen diversity. We also hope that the database will continue to be a valuable tool for identifying characteristics of viruses likely to pose the greatest emerging public health threat. We note that the database could be extended to include further information on human-infective RNA viruses. We welcome and encourage suggestions from the scientific community for updating the information contained in the database, with the proviso that all information included must be supported by a published literature reference.

## Methods

Literature searches were performed on multiple occasions (usually annually) over a period of 18 years up to July 2017. Any inconsistencies or ambiguities in the interpretation of the literature were resolved by reaching consensus following independent assessments by at least two individuals (always including one or both authors). Throughout, a positive data entry implies positive evidence from the scientific literature; a negative data entry may reflect a lack of information or partial information and it is to be expected that some negative data entries will be revised as further studies are published.

No new code was used in compiling this database.

### Identification of human-infective species

Viruses were eligible for inclusion in our catalogue only if they were classified as species by the ICTV as of July 21st 2017. The main ICTV criteria for defining RNA virus species are: relatedness based on sequence data; serological cross-reactivity; host range; and transmission route. We note that our focus on species ignores pertinent within-species variation. For example, there is huge variation in the epidemiology of subtypes of *Influenza A virus*: contrast the human influenza viruses such as H1N1 and H3N2 with avian influenza viruses such as H5N1 and H7N9 that only occasionally infect humans. Although *Influenza A virus* is exceptionally variable in this respect, there are other virus species with distinct subtypes that differ in traits such as antigenicity (e.g. *Dengue virus*) or host preference (e.g. *Rabies virus*). Here, we combine the traits of all identified subtypes to make up the traits of that virus species as a whole.

Our list of human-infective RNA viruses results from a series of systematic literature reviews carried out over the past 18 years. Literature reviews covered multiple reference databases (Web of Science, Google Scholar, PubMed and Scopus), supplemented by secondary sources such as the WHO and CDC web sites, moderated web resources such as ProMed [www.promedmail.org/] and one comprehensive review article^10^. We also followed up primary references linked to RNA virus genome sequence data in NCBI [www.ncbi.nlm.nih.gov/nucleotide/], where this suggested human infection. Structured searches typically included the following key words: human and virus and (case* or patient* or infection* or disease* or outbreak* or zoono*). Searches for newly reported viruses added the key word term: (new or novel). The critical criteria for inclusion in the database are: i) RNA virus species recognised by the International Committee for the Taxonomy of Viruses (ICTV) as of July 2017); ii) peer-reviewed primary report providing robust evidence of human infection.

Only evidence of infections with the specific virus species, or strains/variants falling under that species were accepted; reports of “[virus name]-like” or “potential [virus name]” infections were excluded. Both natural infections and unintentional iatrogenic or otherwise artificially-acquired infections (e.g. occupational laboratory exposure) were considered as evidence supporting human infection, though intentional infections (e.g. experimental inoculation) and in vitro infections (e.g. cell cultures) were excluded. Acceptance of viruses as human-infective was primarily based on expert review, where the following were systematically taken into consideration: diagnostic method(s) used to identify human infection, number of reported infections, number of authors independently reporting infection, author confidence in identity and infectivity of virus. Pathogenicity was not considered a criterion for human infectivity. Where a virus was only known to infect humans from serological methods, we attempted to minimise false positives from serological cross-reactions with related viruses by only accepting evidence from studies where cross-reactions did not demonstrably occur. However, we have indicated where a virus is known only from serological evidence; there are 36 viruses of this kind, of which 28 have not been reported in humans for the past 10 years.

In summary, it is important to note that the information reported here reflects the published literature rather than direct testing of human infectivity for all virus species listed.

### Discovery date

Dates of first reported human infection are supported by a peer-reviewed primary publication. We treated the date of publication as the date of discovery of human infection and we used structured searches (as above) to confirm the absence of earlier reports. The linked publication provides available details of detection and identification methods used.

### Level of transmissibility

Data on transmissibility in humans are reproduced from a previous study^2^; full details of the search strategy are provided in the Technical Appendix to that publication.

### Host range

Literature reviews for host range data were conducted within Web of Science, Google Scholar, and Scopus. Structured search key words were: (virus name and all synonyms listed by ICTV separated by ‘or’) and (host or range or reservoir or animal or mammal or bird or avian). Data on host range is supported by peer-reviewed primary publications where available (PubMed IDs and hyperlinks provided), supplemented by information from the Global Mammal Parasite Database^11^ and one primary publication^12^. Our data were supplemented by reference to an extensive data set on virus-mammal associations provided prior to publication by Kevin Olival of EcoHealth Alliance in New York; these data have since been published^13^.

### Transmission route

Literature reviews for transmission route data were conducted within Web of Science, Google Scholar, and Scopus. Structured search key words were: (virus name and all synonyms listed by ICTV separated by ‘or’) and (transm* or *borne or vector). Information on transmission routes is similarly supported by peer-reviewed primary publications where available (PubMed IDs and hyperlinks provided).

## Data Records

The database is available via Edinburgh DataShare in. xslx format (Data Citation 1) and contains the following records for all 214 currently recognised species of RNA virus for which there is published evidence of human infectivity.

Column A: Current ICTV species name. Human-infective RNA viruses identified as described above.

Column B: Current ICTV genus name. From the ICTV website (https://talk.ictvonline.org/).

Column C: Current ICTV family name. From the ICTV website (https://talk.ictvonline.org/).

Column D: Whether or not the virus is enveloped (1, 0). From the ICTV website (https://talk.ictvonline.org/).

Column E: Genome type (5 types: negative, positive or ambisense single-stranded RNA; double stranded RNA; single-stranded reverse transcriptase RNA).

Column F: ICTV taxonomic history URL (ICTV taxon ID and hyperlink to corresponding webpage).

Column G: Discovery date as calendar year of first published report of virus infecting humans. Obtained from literature review as described above.

Column H: Discovery reference (PubMed ID where available and hyperlink to reference).

Column I: Whether or not virus is known only from serological evidence (Y, N).

Columns J to R: Whether or not transmission to humans is via any of nine possible routes (1, 1*, 0 where 1* indicates inferred from closely related species or assumed to be the same as the animal-to-animal route). NB. Vector=arthropod vector; Broken skin includes wounds and bites; Maternal=mother to offspring transmission in utero or via breast milk; Direct contact=any form of close physical contact. Obtained from literature review as described above.

Columns S, T: Transmission route references (PubMed ID and hyperlink to reference(s)).

Column U: Transmission level as 2 (=human infective but not transmissible between humans); 3 (=transmissible from one human to another by any natural route including arthropod vectors, but restricted to self-limiting outbreaks); 4a (=endemic in human populations or have the potential to cause major epidemics, and are known to naturally infect non-human hosts); or 4b (=endemic in human populations or have the potential to cause major epidemics, and are only known to infect humans (ignoring arthropod vectors)). Obtained from literature review as described above.

Column V: Whether or not there is evidence of person-to-person transmission (1, 0). Obtained from Column U (1=level 3, 4a or 4b; 0=level 2).

Column W: Host range summary as broad (=humans and at least one other non-primate host category), narrow (=humans only or humans and other primates only) or unknown.

Columns X to AC: Whether or not the virus is known to be capable of infecting any of six host categories (1, 0): only humans; or humans plus non-human primates, other mammals, birds, reptiles or fish. Obtained from literature review as described above.

Columns AD, AE: Host range references (PubMed ID and hyperlink to reference(s)).

## Technical Validation

Information on discovery date, transmissibility level, host range and transmission route is all supported by a reference from the scientific literature. Links to the supporting literature references are provided as the PubMed ID if available or other reference database if not (hyperlinks included in the database columns H, S, T, AD and AE). Over 90% of discovery dates are supported by PubMed linked reference.

## Usage Notes

The database contains no information requiring restrictions on use. The database is licenced as Creative Commons Attribution 4.0.

## Additional information

**How to cite this article**: Woolhouse, M. E. J. & Brierley, L.Epidemiological characteristics of human-infective RNA viruses *Sci. Data* 5:180017 doi: 10.1038/sdata.2018.17 (2018).

**Publisher’s note**: Springer Nature remains neutral with regard to jurisdictional claims in published maps and institutional affiliations.

## Supplementary Material



## Figures and Tables

**Table 1:** Numbers of RNA virus species that exhibit specified levels of transmissibility in human populations (fully defined in the main text).

Level of transmissibility	No. species
2 (not transmissible)	123
3 (self-limiting outbreaks)	31
4a (human virus, also zoonotic)	34
4b (human virus, not zoonotic)	26

**Table 2:** Numbers of RNA virus species naturally infecting additional host categories. Note that many viruses infect more than one host category.

Host category	No. species
Humans only	26
Non-human primates	61
Other mammals	158
Birds	37
Reptiles	7
Fish	1

**Table 3:** Numbers of RNA virus species with specified routes of transmission to humans. Note that many viruses are transmitted by more than one route.

Transmission route	No. species
Vector-borne	91
Inhalation	67
Ingestion	33
Fomites	19
Sexual	10
Broken skin	25
Iatrogenic	9
Maternal	28
Close physical contact	68
